# Feedback of coastal marshes to climate change: Long‐term phenological shifts

**DOI:** 10.1002/ece3.5215

**Published:** 2019-06-13

**Authors:** Yu Mo, Michael S. Kearney, R. Eugene Turner

**Affiliations:** ^1^ Department of Environmental Science and Technology University of Maryland College Park Maryland; ^2^ Department of Oceanography and Coastal Sciences Louisiana State University Baton Rouge Louisiana

**Keywords:** blue carbon, coastal marshes, nonlinear mixed model, phenology, vegetation–climate feedback

## Abstract

Coastal marshes are important carbon sinks facing serious threats from climatic stressors. Current research reveals that the growth of individual marsh plants is susceptible to a changing climate, but the responses of different marsh systems at a landscape scale are less clear. Here, we document the multi‐decadal changes in the phenology and the area of the extensive coastal marshes in Louisiana, USA, a representative of coastal ecosystems around the world that currently experiencing sea‐level rise, temperature warming, and atmospheric CO_2_ increase. The phenological records are constructed using the longest continuous satellite‐based record of the Earth's ecosystems, the Landsat data, and an advanced modeling technique, the nonlinear mixed model. We find that the length of the growing seasons of the intermediate and brackish marshes increased concomitantly with the atmospheric CO_2_ concentration over the last 30 years, and predict that such changes will continue and accelerate in the future. These phenological changes suggest a potential increase in CO_2_ uptake and thus a negative feedback mechanism to climate change. The areas of the freshwater and intermediate marshes were stable over the period studied, but the areas of the brackish and saline marshes decreased substantially, suggesting ecosystem instability and carbon storage loss under the anticipated sea‐level rise. The marshes' phenological shifts portend their potentially critical role in climate mitigation, and the different responses among systems shed light on the underlying mechanisms of such changes.

## 1 INTRODUCTION

Coastal marsh carbon is an important component of the global carbon budget (Duarte, Losada, Hendriks, Mazarrasa, & Marbà, 2013). Coastal marshes have high primary production, efficiently trap suspended organic carbon when flooded, and undergo slow carbon decomposition rates under anaerobic conditions (McLeod et al., 2011; Nellemann & Corcoran, 2009). The amount of carbon stored per unit area in stable coastal marshes can be far greater than that of forests, and the carbon stored may remain for millennia, as compared to decades or centuries in forests (Nellemann & Corcoran, 2009). The marshes and their carbon stocks, however, are susceptible to the direct and indirect effects of climate change (Hinson et al., 2017; Nahlik & Fennessy, 2016). Depending on the marshes' vegetation composition, the current ambient conditions, and other environmental factors such as nutrients, the marsh plant growth, and the resulting primary production can be stimulated or hindered by temperature warming, sea‐level rise, and elevated atmospheric CO_2_ concentration (Charles & Dukes, 2009; Erickson, Megonigal, Peresta, & Drake, 2007; Langley & Megonigal, 2010; Langley, Mozdzer, Shepard, Hagerty, & Megonigal, 2013; Morris, Sundareshwar, Nietch, Kjerfve, & Cahoon, 2002). The decomposition of marsh substrates and hence the carbon sequestration rate can be promoted or inhibited by saltwater intrusion and global warming, varying among different marsh systems and being controlled by concurrent stressors (Craft, 2007; Wu, Huang, Biber, & Bethel, 2017). Given the complex and dynamic outcomes of the various factors, it is hard to predict how coastal marshes will respond to the simultaneous climatic stresses at a landscape scale. Yet, this is a key piece of information needed in order to assess ecosystem sustainability and to predict future changes.

Here, we study the multi‐decadal phenology of four distinct coastal marsh systems to gain insights into their ability to uptake carbon in relation to climate change at a broad scale (Walther et al., 2002). We use the longest continuous satellite‐based record of the Earth's ecosystems, the Landsat Climatic Data Records (CDRs) and an advanced modeling technique, the nonlinear mixed model, to reconstruct the phenology between 1984 and 2014 for the different coastal marsh systems in Louisiana, USA, one of world's largest coastal marsh habitats that share the same climatic stressors as many other coastal ecosystems around the world. We speculate that climate change (i.e., temperature, sea‐level, and atmospheric CO_2_ concentrations) has influenced the phenology of the marshes at the ecosystem level and that the influence is a function of whether the marshes are tidal freshwater, intermediate, brackish, or saline systems. We also hypothesize that the climatic influences will continue, if not increase, under future climate scenarios.

## 2 METHODS

### 2.1 Study area

**Figure 1:**
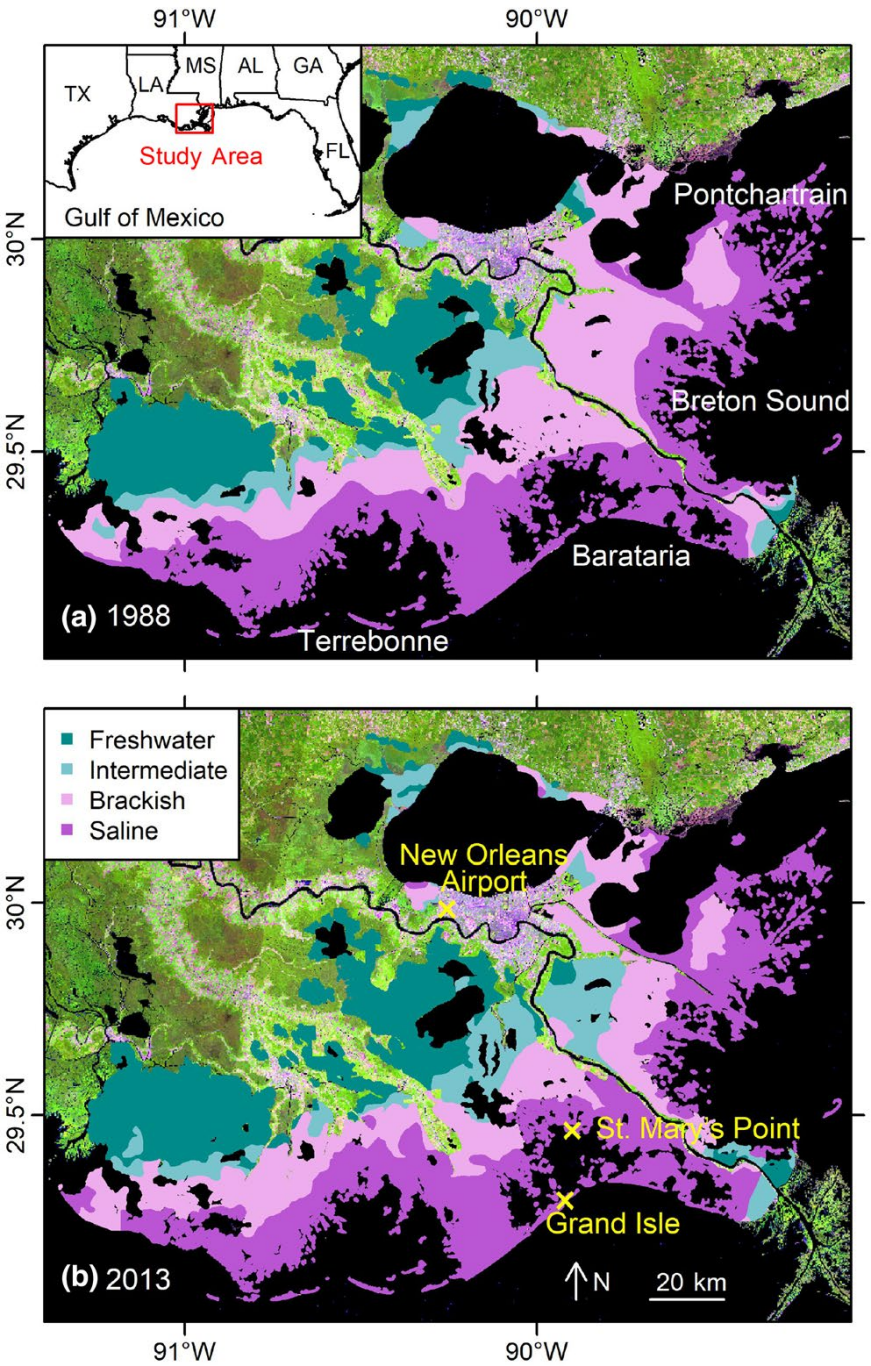
The distribution of freshwater (*dark green areas*), intermediate (*light green areas*), brackish (*light purple areas*), and saline (*dark purple areas*) marshes in the study area—four major basins in Louisiana, USA, at the northern Gulf of Mexico, that is, the Barataria, the Breton Sound, the Pontchartrain, and the Terrebonne basins—in 1988 (Panel a) and 2013 (Panel b). The locations of the Grand Isle station, the New Orleans Airport station, and the St. Mary's Point station are also shown (*yellow crosses*) in Panel b

The study area is in four major basins in Louisiana, USA, at the northern Gulf of Mexico: the Barataria, Breton Sound, Pontchartrain, and Terrebonne basins (Figure 1). The marshes are classified into freshwater, intermediate, brackish, and saline systems based on vegetation association and salinity (Gosselink, 1984). Freshwater marshes occupy habitats with a salinity <0.5 ppt and are dominated by *Panicum hemitomon*,* Sagittaria falcata*, and *Eleocharis* sp. Intermediate marshes occupy habitats with salinity from 0.5 to 5 ppt and are dominated by *Spartina patens* and *Phragmites australis*. Brackish marshes are characterized by salinities ranging from 5 to 18 ppt, and the dominant species are *Spartina patens* and *Distichlis spicata*. Saline marshes occur where salinity is >18 ppt, and the dominant species are *Spartina alterniflora*,* Distichlis spicata*, and *Juncus roemerianus*. The percentage of C4 plants increases with higher salinities, whereas the species richness and diversity decrease. The boundaries of the different marshes are obtained from the United States Geological Survey (USGS) vegetative survey for Louisiana coastal marshes (Chabreck & Linscombe, 1988, 1997; Linscombe & Chabreck, 2001; Sasser, Visser, Mouton, Linscombe, & Hartley, 2008, 2014).

### 2.2 Marsh phenology modeling

We use the longest continuous satellite record of the Earth's ecosystems, the Landsat CDRs from 1984 to 2014, to create the phenological records of the marshes (Table A1). The data are collected by three Landsat series satellites: (a) the Landsat 5 equipped with the Thematic Mapper (TM) that launched in 1984 and decommissioned in 2011; (b) the Landsat 7 equipped with the Enhanced Thematic Mapper Plus (ETM+) that launched in 1999; and (c) the Landsat 8 equipped with the Operational Land Imager (OLI) that launched in 2013. All three sensors have a 30‐m spatial resolution and a 16‐day temporal revisit cycle. The study area locates within the Landsat scenes of Path 22 Row 40 and Path 22 Row 39. There are 359 relatively cloudless images (mosaics of the two Landsat scenes) used for the phenology modeling.

The Normalized Difference Vegetation Index (NDVI) values are calculated as a proxy for the marshes' aboveground biomass (*R* ^2^ = 0.7; Mo, Kearney, Riter, Zhao, & Tilley, 2018), and the NDVI‐based phenological records of the marshes are modeled using an advanced modeling technique, the nonlinear mixed model (Mo, Momen, & Kearney, 2015). This method is developed by Mo et al. (2015) to provide a rigorous statistical analysis for phenological curves of different vegetation that are represented by nonlinear functions with repeated‐measure variables. Phenological measurements (i.e., the NDVI) made on the same observational units (i.e., marsh systems) over time are treated as repeated measurements. The phenological records of the different marsh systems are fitted into three nonlinear models, the Gaussian, the stepwise Gaussian, and the stepwise logistic functions. The goodness‐of‐fit of the models is assessed via the Efron's pseudo *R* ^2^ and the Akaike Information Criterion (AIC), the Akaike Information Criterion Correction (AICC), and the Bayesian Information Criterion (BIC). The pseudo *R* ^2^, a statistic similar to *R* ^2^ in the linear regression, indicates the percent variance explained by the nonlinear models (Hardin, Hilbe, & Hilbe, 2007). The pseudo *R* ^2^ ranges from −∞ to 1. A pseudo *R* ^2^ closer to 1 indicates more variability in the data is explained. The AIC, AICC, and BIC indices evaluate models based on the principle of parsimony, that is, a model explains more variation in the data with fewer variables is considered a better fit (Boyce, Vernier, Nielsen, & Schmiegelow, 2002; Richards, 2005). Key phenological parameters, that is, peak NDVI, peak NDVI day, and growing season length (bracketing days that had NDVI >90% of peak NDVI) for each marsh system in each year are estimated from the best‐fit model. The phenology modeling only considered existing marshes, that is, it is corrected for the marsh area changes over the 30 years.

### 2.3 Marsh area estimation

The areas of the freshwater, intermediate, brackish, and saline marshes are estimated using cloud‐free Landsat 5 TM and Landsat 8 OLI data. The marshes type boundaries are determined using the USGS vegetative survey for Louisiana coastal marshes done in 1988, 1997, 2001, 2007, and 2013 (Chabreck & Linscombe, 1988, 1997; Linscombe & Chabreck, 2001; Sasser et al., 2008, 2014). The marshland area within the boundaries is estimated using the C version of the Function Mask (CFMask) that comes with the Landsat CDRs (Zhu & Woodcock, 2012). There are 2, 6, 5, 4, and 1 mosaic images used for 1988, 1997, 2001, 2007, and 2013, respectively, for a total of 18 images (Table A2). The overall accuracy of using the CFMask for estimating the marshland area is 0.89 ± 0.04 (verified using the USGS Digital Orthophoto Quadrangle, DOQ; Table A3).

### 2.4 Climatic and environmental data

We acquire records of the atmospheric CO_2_, air temperature, Oceanic Niño Index (ONI), sea‐level, and salinity of the study area from stations of different US Federal and State agencies. The annual mean sea‐level data are from the National Oceanic and Atmospheric Administration (NOAA) station # 8761724 Grand Isle, Louisiana, USA (Tides and Currents). The annual mean sea‐level are calculated from the monthly means. The atmospheric CO_2_ records are from the NOAA Carbon Cycle Cooperative Global Air Network Niwot Ridge Station, Colorado, USA (Earth System Research Laboratory). The National Weather Service (NWS) #12916 New Orleans Airport Station, Louisiana, USA, is the source for the precipitation and temperature data (Climatological Data Publications). The ONI records are obtained from the NWS Climate Prediction Center (Cold & Warm Episodes by Season). The Louisiana Department of Wildlife and Fisheries station #317/USGS station #07380251 St. Mary's Point, Barataria Bay, Louisiana, USA, provides the salinity data (National Water Information System), and the annual means are calculated from the daily measurements. The correlations between the marsh phenology and marsh area with the climatic and environmental factors are analyzed using the pairwise Pearson's correlation analysis.

### 2.5 Phenology prediction

**Figure A1:**
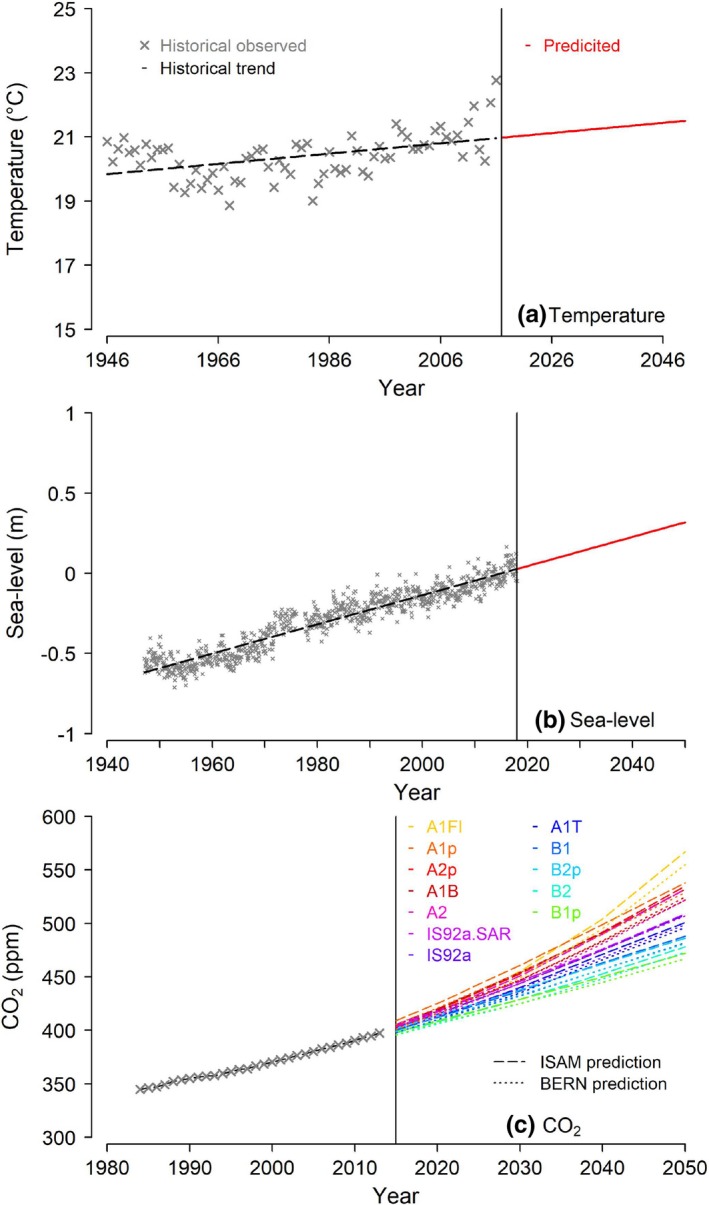
Panel (a) shows the air temperature at the National Weather Service (NWS) #12916 New Orleans Airport station, Louisiana, USA, since 1946 (*gray crosses*) and its trend (*black dash line*) and its projection until 2050 (*red solid line*). Panel (b) shows the sea‐level at the National Oceanic and Atmospheric Administration (NOAA) # 8761724 Grand Isle station, Louisiana, USA, since 1947 relative to the most recent National Tidal Datum Epoch (*gray crosses*) and its trend (*black dash line*) and projection until 2050 (*red solid line*). Panel (c) shows the atmospheric CO_2_ records from the NOAA Carbon Cycle Cooperative Global Air Network Niwot Ridge Station, Colorado, USA since 1984 (*gray crosses*) and its trend (*black dash line*) and projections (*rainbow color lines*). The future atmospheric CO_2_ atmospheric concentrations are obtained from the Intergovernmental Panel on Climate Change Fourth Assessment Report (IPCC AR4). The projections are from two carbon cycle models (i.e., the Bern model, or BERN,* dotted line*, and the Integrated Science Assessment Model, or ISAM,* dash line*) under different emission scenarios (i.e., A1B, A1T, A1FI, A2, B1, B2, A1p, A2p, B1p, B2p, IS92a, IS92a/SAR)

Linear models describing the correlations between the marsh phenology and the climatic variables are built on the historical data and used to predict the marsh phenology in the future (until 2050). The future sea‐level and air temperature in the study area are estimated using linear models based on the sea‐level data from the NOAA Grand Isle Station, and the air temperature data from the NWS New Orleans Airport Station, both dating back to the 1940s (Figure A1a, b). The slope of the sea‐level increase is 9 mm/year (*p* < 0.01), which is in consistence with the literature (González & Törnqvist, 2011; Jankowski, Törnqvist, & Fernandes, 2017). The temperature increases at a speed of 0.016°C/year (*p* < 0.01), falling within the lower ranges of projections from the Fourth Assessment Report of the Intergovernmental Panel on Climate Change (IPCC AR4, 2007). The ranges of possible future atmospheric CO_2_ concentrations are obtained from the IPCC AR4 (Data Distribution Centre). The future atmospheric CO_2_ concentrations are from two carbon cycle models (i.e., the Bern model, or BERN, and the Integrated Science Assessment Model, or ISAM) and under different emission scenarios (i.e., A1B, A1T, A1FI, A2, B1, B2, A1p, A2p, B1p, B2p, IS92a, and IS92a/SAR; Figure A1c).

## 3 RESULTS

### 3.1 Marsh phenological changes

**Figure 2:**
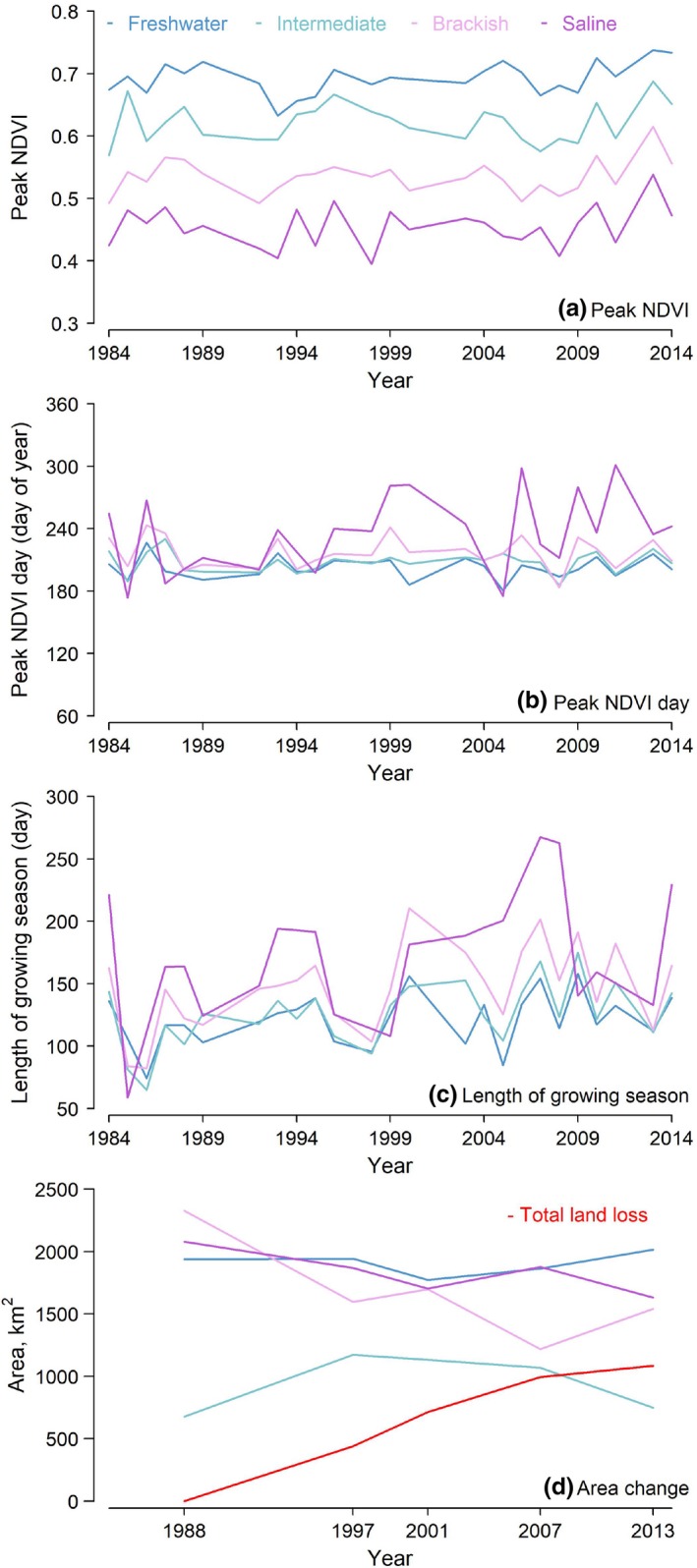
Phenology and areas of the coastal freshwater (*dark green*), intermediate (*light green*), brackish (*light purple*), and saline (*dark purple*) marshes in Louisiana from 1984 to 2014. The three key phenological parameters estimated are peak NDVI (Panel a), peak NDVI day (Panel b), and growing season length (bracketing days that had NDVI greater than 90% of peak NDVI; Panel c). The marsh areas are estimated in 1988, 1997, 2001, 2007, and 2013 (Panel d).

The NDVI‐derived phenological records of the freshwater, intermediate, brackish, and saline marsh systems from 1984 to 2014 are well‐described by our models (pseudo‐*R* ^2^ 0.86 ± 0.11; Table 1). Exceptions are years when not enough relatively cloudless images were collected, and thus the phenological parameters of the respective marsh units in those years cannot be estimated: that is, all marshes in 1990, 1991, 1997, 2001, 2002, and 2012 and the saline marshes in 1994 and 1998. The phenology of the different marsh systems varies. The freshwater marshes had the highest annual peak NDVI (around 0.7), followed by the intermediate, brackish, and saline marshes (around 0.6, 0.5, and 0.4, respectively; Figure 2a). The NDVI of the freshwater, intermediate, and brackish marshes peaked between July and August, while the NDVI of the saline marshes peaked between July and October (Figure 2b). The length of the growing seasons of the freshwater, intermediate, brackish, and saline marshes varied between 2 and 8 months among different marsh systems and years (Figure 2c).

**Table 1:** The pseudo R^2^, the Akaike Information Criterion (AIC), the Akaike Information Criterion Correction (AICC), and the Bayesian Information Criterion (BIC) of the best‐fit phenological model (i.e., the Gaussian, G; the stepwise Gaussian, SG; or the stepwise logistic, SL, function) of the freshwater, intermediate, brackish, and saline marshes from 1984 to 2014 **(**Exceptions are years when not enough cloudless images were collected, including all marshes in 1990, 1991, 1997, 2001, 2002, and 2012 and saline marshes in 1994 and 1998. The phenological parameters of the respective marsh units in the years cannot be estimated and are not shown in the table)

Year	Best‐fit model	AIC	AICC	BIC	Pseudo R^2^			
					Freshwater	Intermediate	Brackish	Saline
1984	G	‐322.7	‐317.0	‐311.3	0.97	0.99	0.91	0.57
1985	G	‐264.7	‐258.2	‐253.3	0.96	0.97	0.90	0.57
1986	SG	‐241.5	‐231.0	‐227.3	0.94	0.89	0.89	0.91
1987	G	‐360.1	‐356.2	‐348.7	0.97	0.95	0.93	0.85
1988	G	‐428.4	‐423.8	‐417.1	0.94	0.94	0.83	0.61
1989	G	‐224.5	‐217.2	‐213.2	0.90	0.93	0.83	0.81
1992	G	‐373.3	‐367.9	‐362.0	0.92	0.93	0.93	0.92
1993	G	‐397.3	‐393.3	‐384.9	0.90	0.93	0.95	0.88
1994	G	‐358.7	‐354.1	‐346.3	0.87	0.88	0.82	‐
1995	G	‐459.2	‐455.7	‐446.8	0.95	0.96	0.93	0.80
1996	G	‐346.3	‐342.7	‐333.9	0.89	0.89	0.87	0.71
1998	G	‐498.3	‐494.6	‐485.9	0.98	0.96	0.94	‐
1999	G	‐918.2	‐916.4	‐905.9	0.94	0.89	0.87	0.89
2000	G	‐934.7	‐932.9	‐922.3	0.91	0.84	0.63	0.58
2003	G	‐794.3	‐792.0	‐781.9	0.86	0.91	0.86	0.80
2004	G	‐534.6	‐531.5	‐522.3	0.89	0.94	0.91	0.77
2005	SG	‐1173	‐1170	‐1157	0.94	0.92	0.91	0.83
2006	G	‐790.0	‐788.1	‐777.7	0.92	0.95	0.90	0.86
2007	G	‐654.3	‐651.7	‐642.0	0.85	0.82	0.75	0.56
2008	G	‐881.0	‐879.1	‐868.6	0.92	0.81	0.74	0.51
2009	G	‐861.8	‐859.9	‐849.4	0.94	0.91	0.85	0.78
2010	G	‐670.8	‐668.8	‐658.5	0.94	0.92	0.89	0.93
2011	G	‐1102	‐1101	‐1090	0.88	0.76	0.66	0.58
2013	G	‐510.5	‐507.5	‐498.2	0.99	0.99	0.96	0.87
2014	G	‐981.4	‐979.8	‐969.1	0.91	0.89	0.84	0.66

The local sea‐level, temperature, and CO_2_ all rose from 1984 to 2014 (*p* < 0.05) (Figure A1). The phenology of the different systems varied in response to the climate conditions (Table A4). The peak NDVI values of all four marsh systems did not significantly change over time. The peak NDVI day of the saline marshes showed no significant changes over time but correlated with air temperature (*r* = 0.5, *p* < 0.05). The length of the growing seasons of the intermediate and brackish marshes significantly increased (by 36.4 and 38.0%, respectively; *p* < 0.05 in both cases), and positively correlated with variations in the atmospheric CO_2_ (*r* = 0.4 and *p* < 0.05 in both cases).

### 3.2 Marsh area changes

**Figure 3:**
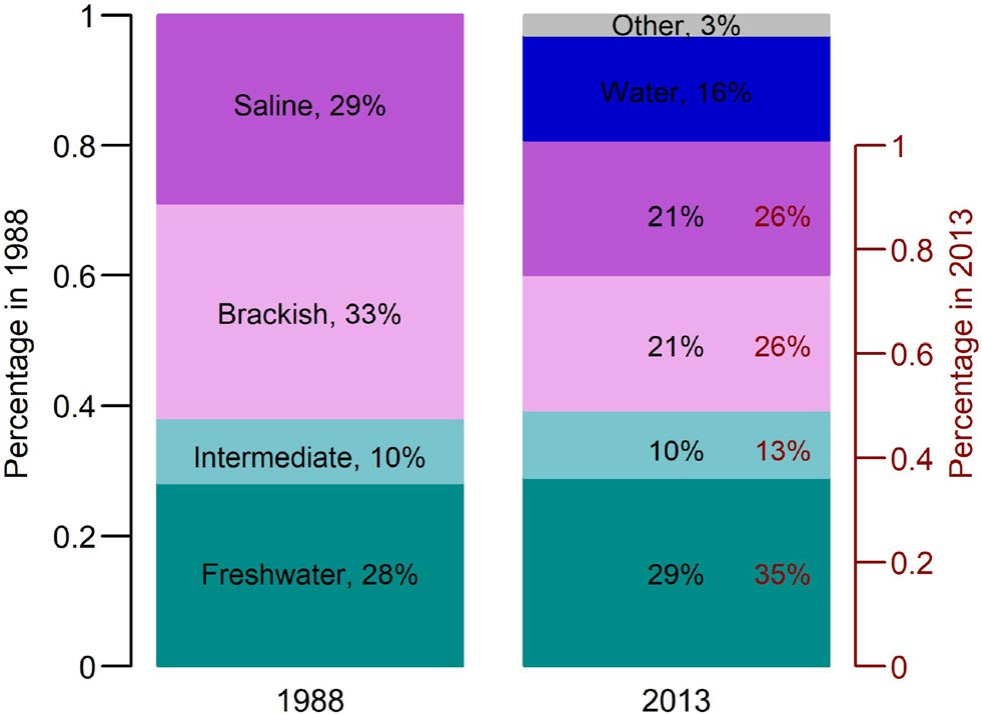
The percentage areas of the freshwater (*dark green*), intermediate (*light green*), brackish (*light purple*), and saline (*dark purple*) marshes, water (*blue*), and other landcover type (*gray*) in the study area in 1988 (left axis, *black numbers*) and 2013 (right axis, *red numbers*)

The total marsh area in 2013 was about 80% of the total marsh area in 1988, and 16% of the marshes in 1988 turned into water by 2013 (Figure 3). In 1988, the freshwater, intermediate, brackish, and saline marshes composed 28%, 10%, 33%, and 29% of  the marshes in the study area, respectively, and 35%, 13%, 26%, 26% in 2013. The areas of the freshwater and intermediate marshes were quite stable, and their increased percentage was mostly a result of the decrease of the total marsh area. The marsh loss mainly occurred in the brackish and saline marshes, contributing to most of the 16% change of marsh‐to‐water from 1988 to 2013. The decrease of the brackish and saline marshes' areas were significant over time (*p* < 0.05 in both cases; Figure 2d). The area of the brackish marshes decreased for 52.9% (from 2,326 km^2^ in 1988 to 1,541 km^2^ in 2013), and the area of the saline marshes decreased for 17.9% (from 2,080 to 1,631 km^2^). The area of the brackish marshes was negatively correlated with sea‐level, CO_2_ concentration, and temperature, and positively with precipitation (*r* = −0.8, −0.7, −0.9, and 0.7, respectively; *p* < 0.05 in all cases; Table A4); the area of the saline marshes was negatively correlated with sea‐level rise rates and CO_2_ concentration (*r* = −0.5 and −0.6, respectively; *p* < 0.05 in both cases; Table A4).

The area changes of the different marsh systems from 1988 to 2013 varied spatially (Figure 1). There was an expansion of brackish marshes into intermediate marshes and saline marshes in the Terrebonne Basin and a replacement of intermediate marshes with saline marshes in the Barataria Basin. Brackish marshes expanded into intermediate and freshwater marshes in the Breton Sound Basin.

### 3.3 Future marsh phenology

In Section 3.1, we find that the peak NDVI day of the saline marshes was significantly correlated with air temperature and that the length of the growing seasons of the intermediate and brackish marshes was significantly correlated with atmospheric CO_2_ (*p* < 0.05 in all cases). Based on these correlations, we use the phenological and environmental data from 1984 to 2014 to build models to predict the marshes' future phenology. The model for predicting the brackish marsh growing season length (day) is −171.0538 + 0.8606 × CO_2_. The model for predicting the intermediate marsh growing season length (day) is −139.3226 + 0.7169 × CO_2_. The model for predicting the saline marsh peak NDVI day (day of year) is −376.507 + 29.365 × Temperature.

**Figure 4:**
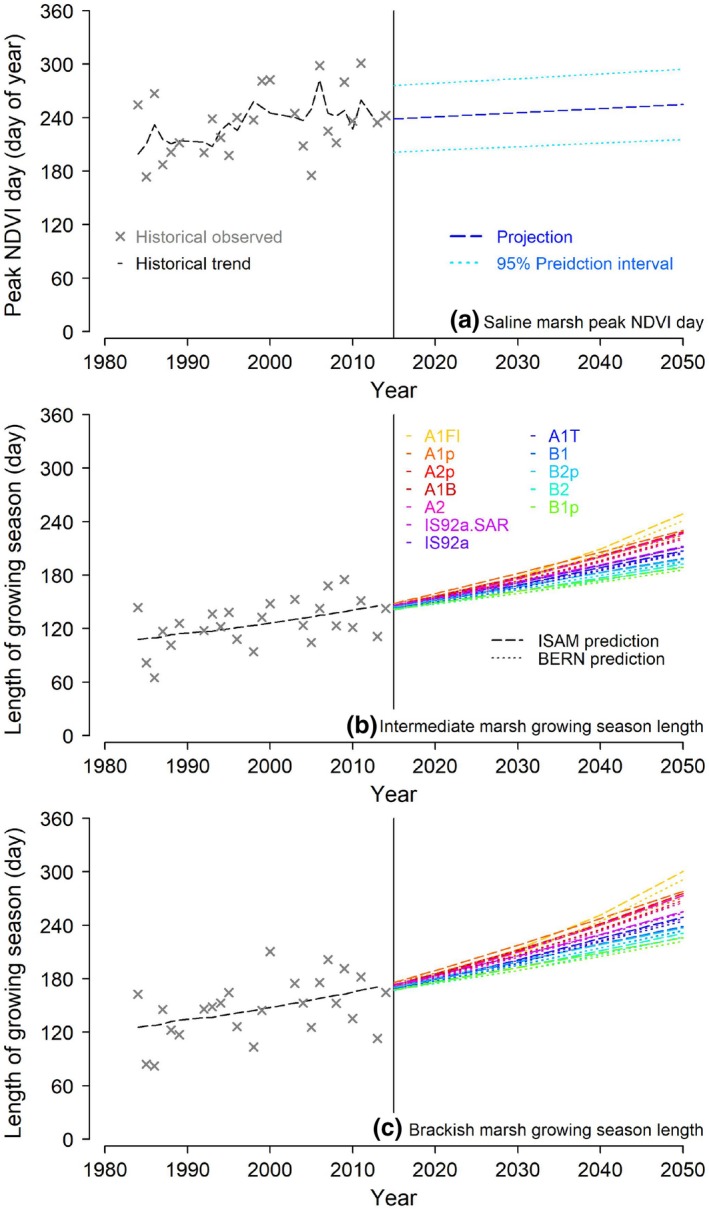
Historical (1984‐2014) and predicted marsh phenology (till 2050) under future temperature and CO_2_ projections. The future air temperature in the study area is predicted using a linear model based on the air temperature from the National Weather Service New Orleans airport station, Louisiana, USA, since the 1940s. The future atmospheric CO_2_ concentrations are estimations from two carbon cycle models (i.e., the Bern model, or BERN, and the Integrated Science Assessment Model, or ISAM) under the different emission scenarios reported in the Intergovernmental Panel on Climate Change Fourth Assessment Report (IPCC AR4)

The changes in the peak NDVI day for saline marshes will continue at the same rate (Figure 4a). The peak NDVI day of the saline marshes moved from July to August during the past 30 years and is projected to be in September in 2050. It should be noted that based on our analysis, the changes of the peak NDVI day of saline marshes were not directly correlated to time, but were correlated to air temperate in the study area which increases over time. The changes in the brackish and intermediate marshes' growing season length will accelerate under all CO_2_ emission scenarios tested (Figure 4b,c). From 1984 to 2014, the length of the growing seasons of the intermediate and brackish marshes increased for around 40 and 50 days, at rates of 1.3 and 1.6 days/year, respectively. Under the A1F1 scenario (a future world of very rapid economic growth with fossil intensive energy sources) with the ISAM, the growing seasons of the intermediate and brackish marshes will lengthen at the highest rates, for around 140 and 175 days in the next 30 years, equals to 4 and 5 days/year, respectively. Under the B1p scenario (a convergent world with the same global population) with the BERN, the length of the growing seasons of the intermediate and brackish marshes will increase at the lowest rates—but still faster than the past 30 years—for around 80 and 90 days in the next 30 years, equals to 2.2 and 2.7 days/year, respectively. It should be noted that these predictions rely solely on air temperature and CO_2_ emissions scenarios and do not take nutrient limitations or other limiting factors into account.

## 4 DISCUSSION

### 4.1 The marshes' phenological changes in the last 30 years

The correlations between the growing season length and the atmospheric CO_2_ concentration may be the result of the stimulation of elevated CO_2_ concentration on photosynthesis in marsh plants (Cherry, McKee, & Grace, 2009; Rasse, Peresta, & Drake, 2005). This is the first study we know of that reports a positive correlation between atmospheric CO_2_ concentration and coastal marshes' growing season length, which reflects a broad pattern of ecosystem change due to a changing climate (Walther et al., 2002). The various responses among marsh systems are likely to reflect the different physiological characteristics of the marsh plants. The intermediate and brackish marshes have a high percentage of C3 plants, whereas saline marshes are mostly C4 plants. Elevated atmospheric CO_2_ promotes the plant growth of C3 marsh species, for example, *Schoenoplectus americanus*,* Scirpus maritimus*,* Scirpus olneyi*, and *Puccinellia maritima*, but does not enhance, or even impairs, the plant growth of C4 species, for example, *Distichlis spicata*,* Spartina alterniflora*, and *Spartina patens* (Arp, Drake, Pockman, Curtis, & Whigham, 1993; Cherry et al., 2009; Drake, 2014; Erickson et al., 2007; Rasse et al., 2005). This is because the increased atmospheric CO_2_ stimulates photosynthesis of C3 plants, but not the photosynthesis of C4 plants that is nearly saturated under ambient conditions as C4 plants concentrate CO_2_ at the site with their primary CO_2_‐fixing enzyme. Moreover, the elevated CO_2_ may even inhibit the plant growth of C4 species by reducing their stomatal conductance, transpiration, and ion uptake (Ghannoum, 2009; Rozema et al., 1991). Yet, the freshwater marshes, which have the highest percentage of C3 plants among the different marsh systems, demonstrate no response to higher CO_2_ levels in our study. One possible explanation is that the high nutrient loading in freshwater marshes—freshwater marshes are closest to the surface runoff—favors species that are unresponsive to elevated atmospheric CO_2_ (Langley & Megonigal, 2010; Langley et al., 2013).

Global warming has a general effect of promoting plant growth that is manifested in increasing the growing season NDVI and lengthening the active growth season, especially in the middle and high latitudes (Myneni, Keeling, Tucker, Asrar, & Nemani, 1997; Zhou et al., 2001). Although it was reported that warming increased the annual peak biomass of the marshes in Massachusetts, USA (Charles & Dukes, 2009), this study did not find a similar effect of temperature on the peak NDVI nor on the growing season length of the marshes in Louisiana. This result might be expected because the ambient temperatures in Louisiana (at latitudes around 30ºN) are already close to the optimum conditions for the marsh growth.

Indeed, the linear models used in this study are highly simplified. These models do not consider other environmental factors, such as water temperature and nutrient availability, which can vary substantially with in situ conditions, or the nonlinear effects and the interactions among the different factors. For instance, the enhancement of CO_2_ uptake by C3 plants may be eventually slowed down by photosynthetic downregulation, nutrient limitations, or increased disturbance from sea‐level rise, thus the CO_2_ fertilization on the marshes will not continue over long time frames as decades (Erickson et al., 2007; Langley et al., 2013). In addition, the marshes may suffer from other stresses such as pests, herbivores, and pathogens that can also be intensified by a warmer climate (Van der Putten, Macel, & Visser, 2010). Nevertheless, this study provides a “baseline” scenario for future marsh phenology with unconstrained sole impacts from temperature or CO_2_, as well as observational inputs for more advanced modeling studies.

### 4.2 The marsh area changes in the last 30 years

Couvillion et al. reported that 25% of Louisiana's coastal marshes that existed in 1932 had been lost by 2010 (Couvillion et al., 2011), and this study shows that 20% of the marshes in 1988 were lost by 2013. These findings document a highly vulnerable ecosystem. Recent studies have found strong correlations between marsh area loss and several climatic variables (Turner, Kearney, & Parkinson, 2017; Turner, Baustian, Swenson, & Spicer, 2006; Kearney & Turner, 2015). In this study, we further the research by separating the area changes of different marsh systems. We find that the areas of the freshwater and intermediate marshes were quite stable; while the area of the brackish and saline marshes decreased significantly and were negatively correlated with sea‐level and CO_2_ concentration. Indeed, the area changes of the Louisiana coastal marshes are likely to relate with both climatic and anthropogenic factors, but here we focus on the climatic factors.

Sea‐level rise may decrease the marsh elevation by reducing their organic accretion (Nyman, Delaune, Roberts, & Patrick, 1993), promoting decomposition of the marsh substrates (Craft, 2007; Weston, Vile, Neubauer, & Velinsky, 2011; Stagg, Schoolmaster, Krauss, Cormier, & Conner, 2017), and increasing erosion (Smith, Cialone, Wamsley, & McAlpin, 2010). Hence, the negative correlation between the marsh area and sea‐level can be expected. On contrary, increased air temperature and CO_2_ concentration can contribute to the marsh stability against sea‐level rise. The elevated air temperature and atmospheric CO_2_ may promote the marsh plant growth (for both aboveground and belowground) and thus their organic accretion (Langley, McKee, Cahoon, Cherry, & Megonigal, 2009; Ratliff, Braswell, & Marani, 2015). Although the enhancement of plant growth from CO_2_, as discussed earlier, is mainly for C3 plants, the fertilization of CO_2_, based on a modeling study, can increase the marsh elevation for a mixed C3 and C4 plant community (by increasing plant production) in similar magnitude to the effect of increasing inorganic sediment input (Ratliff et al., 2015). The maintenance of the marsh elevation can further benefit from the increased belowground biomass: the increased shoot density will enhance the trapping of tidally driven sediment and provide stronger protection against erosion (Temmerman, Moonen, Schoelynck, Govers, & Bouma, 2012; Mudd, D'Alpaos, & Morris, 2010). It should also be noted that the influence of CO_2_ fertilization on marsh elevation is likely to depend on many other factors such as the inorganic sedimentation rate (Langley & Megonigal, 2010; Langley et al., 2009; Ratliff et al., 2015) and may be offset by the enhanced decomposition resulted from a rising air temperature (Charles & Dukes, 2009; Kirwan & Blum, 2011). The fertilization effects of the increased air temperature and atmospheric CO_2_ allow the coastal marshes to be more resilient against sea‐level rise, but this study documents a significant decrease in the brackish and saline marsh systems, where the impact of sea‐level is strongest and more likely to overwhelm the fertilization effects of temperature and CO_2_.

The area changes of the different marsh systems vary spatially. We find an expansion of brackish marshes into intermediate and saline marshes in the Terrebonne Basin, while saline marshes were replaced by intermediate marshes in the Barataria Basin, and brackish marshes were replaced by intermediate and freshwater marshes in the Breton Sound Basin. This study focuses on the temporal changes of the environmental factors over the past decades and only considers the sea‐level and salinity information of one station within the study area. Future studies further examine the spatial pattern of the sea‐level and salinity changes (Jankowski et al., 2017), the relative relationship between sediment supply and sea‐level rise of different locations (Mariotti & Fagherazzi, 2010), and the availability of accommodation space (Schuerch et al., 2018) may provide more insights the spatial patterns of the area changes.

### 4.3 Marshes' feedback to climate change

Regardless of the causes, the increased length of the marshes' growing season has a potentially important impact on the global carbon cycles. The extended growing seasons of the intermediate and brackish marshes directly reflect an increase in aboveground primary production (and possibly an increase in the belowground biomass; Langley et al., 2009). On average, roughly 20% of the marshes net primary production results in carbon storage (Duarte & Cebrian, 1996). The increase in aboveground biomass allows for an increase in photosynthesis and CO_2_ uptake, providing a negative feedback mechanism to the elevated atmospheric CO_2_ and climate change. On the other hand, the loss of the brackish and saline marshes impairs the ecosystems' potential to capture CO_2_ and the stability of the existing carbon storage, which is quite large in the coastal marsh anaerobic substrates (long‐term C accumulation rate at 18‐1713 g C m^−2^ yr^−1^; McLeod et al., 2011; Nahlik & Fennessy, 2016). The stored carbon will be released into the ocean or the atmosphere—which can also be in the form of methane, a more potent greenhouse gas, under the marshes' anaerobic conditions (Whiting & Chanton, 1993)—providing a positive feedback mechanism to climate change. The Louisiana coastal marshes are representatives of coastal ecosystems around the world experiencing various climatic and anthropogenic stressors (Bianchi & Allison, 2009; Wang et al., 2007). This study documents the climate‐driven long‐term phenological shifts of the marshes that, in turn, provide a negative feedback mechanism to the changing climate. A stable coastal marsh system will capture and store more carbon under a changing climate, and compensate, to some extent, for anthropogenic carbon emissions. Such mechanisms highlight the marshes' critical role in climate mitigation and emphasize the importance of the conservation and restoration of coastal ecosystems under the changing climate.

## AUTHORS CONTRIBUTION

Kearney, Mo and Turner conceived and designed the experiments; Kearney and Mo processed and analyzed the data; Kearney, Mo, and Turner wrote the paper.

## Data Availability

Data from this study are publicly available through the Gulf of Mexico Research Initiative Information & Data Cooperative (GRIIDC) at https://data.gulfresearchinitiative.org (https://doi.org/10.7266/n7513w97, 10.7266/N7222RVS, 10.7266/N7X928DB, 10.7266/N71834KW, 10.7266/N7SJ1HPM, 10.7266/N7NS0RZW, 10.7266/N7J10178, 10.7266/N7D798H0, 10.7266/N7WH2N25, 10.7266/N78G8HTQ, 10.7266/N74T6GFT, 10.7266/N7125QRV, 10.7266/N7B56GNT, 10.7266/N7RR1W90, 10.7266/N7W9578Z, 10.7266/N7V98651, 10.7266/N7F47M2T, 10.7266/N7N014MD, 10.7266/N7PN93J0, 10.7266/N7RJ4GJ7, 10.7266/N7MS3QTH, 10.7266/N7H1303W, 10.7266/N7CJ8BK4, 10.7266/N7C827DM, 10.7266/N77S7KVD, 10.7266/N7GX491R).

## APPENDIX 1

### 1.1

**Table A1:** Sampling dates of Landsat imagery (mosaics of Landsat scenes Row 22 Path 39 and Row 22 Path 40) used for the marsh phenology modeling

Year	Day of Year	Satellite	Number of Images
1984	97, 193, 241, 257, 273, 305, 337	Landsat 5	8
1985	19, 83, 99, 211, 243, 323, 339	Landsat 5	7
1986	54, 86, 214, 246, 278, 294, 326	Landsat 5	7
1987	9, 25, 41, 73, 121, 137, 185, 233, 249, 265, 281, 345	Landsat 5	12
1988	28, 44, 76, 124, 140, 172, 252, 284, 300, 316	Landsat 5	10
1989	46, 94, 110, 126, 190, 238, 318, 350	Landsat 5	8
1992	39, 87, 103, 167, 279, 295, 311, 327	Landsat 5	9
1993	25, 73, 105, 137, 153, 201, 217, 233, 249, 265, 361	Landsat 5	11
1994	76, 92, 140, 172, 252, 268, 300, 316, 348	Landsat 5	9
1995	31, 79, 143, 159, 207, 223, 239, 255, 271, 319, 335	Landsat 5	11
1996	18, 82, 98, 130, 146, 162, 178, 194, 210, 306, 338, 354	Landsat 5	12
1998	39, 55, 135, 151, 167, 199, 215, 231, 247, 279, 327	Landsat 5	11
1999	10, 26, 42, 122, 138, 170, 218, 250, 266, 290, 314, 330, 362	Landsat 5	20
	210, 226, 258, 274, 298, 306, 322	Landsat 7	
2000	109, 189, 285, 301, 365	Landsat 5	21
	5, 21, 37, 53, 85, 101, 117, 133, 197, 229, 245, 261, 277, 293, 325, 357	Landsat 7	
2003	5, 21, 69, 117, 149, 197, 245, 261, 277, 293, 349	Landsat 5	16
	13, 221, 285, 317, 333, 365	Landsat 7	
2004	72, 216, 232, 312, 328	Landsat 5	13
	16, 80, 128, 192, 224, 240, 272, 304	Landsat 7	
2005	42, 106, 122, 138, 154, 170, 202, 234, 250, 282, 298, 314	Landsat 5	24
	2, 82, 98, 114, 130, 178, 194, 258, 290, 306, 322, 354	Landsat 7	
2006	13, 45, 61, 173, 269, 285, 301, 317	Landsat 5	21
	5, 85, 117, 133, 165, 181, 197, 229, 245, 293, 309, 325, 357	Landsat 7	
2007	48, 64, 96, 112, 128, 192, 224, 272	Landsat 5	14
	8, 88, 120, 232, 312, 344	Landsat 7	
2008	83, 99, 115, 147, 163, 179, 195, 227, 243, 275, 307, 323	Landsat 5	20
	11, 59, 171, 203, 235, 299, 315, 347	Landsat 7	
2009	21, 37, 149, 181, 213, 245, 293, 309	Landsat 5	19
	13, 29, 61, 77, 93, 157, 173, 237, 285, 317, 365	Landsat 7	
2010	56, 88, 152, 168, 232, 248, 280, 296, 312, 344	Landsat 5	18
	48, 64, 144, 160, 176, 256, 288, 336	Landsat 7	
2011	43, 75, 91, 107, 155, 171, 187, 219, 235, 251, 267, 299, 315	Landsat 5	22
	3, 83, 99, 131, 211, 227, 243, 259, 323	Landsat 7	
2013	24, 72, 88, 200, 280, 296, 312	Landsat 7	14
	144, 176, 240, 256, 272, 288, 352	Landsat 8	
2014	59, 123, 139, 171, 187, 219, 235, 251, 299, 331, 347	Landsat 7	22
	19, 99, 115, 211, 227, 243, 275, 291, 307, 323, 339	Landsat 8	
Total			359

**Table A2:** Sampling dates of Landsat imagery (mosaics of Landsat scenes Row 22 Path 39 and Row 22 Path 40) used for the marsh area estimation

Year	Day of year	Satellite	Total
1988	28,44	Landsat 5	2
1997	20, 36, 244, 276, 308, 340	Landsat 5	6
2001	143, 271, 303, 319, 335	Landsat 5	5
2007	48, 64, 96, 224	Landsat 5	4
2013	352	Landsat 8	1
Total			18

**Table A3:** Confusion matrix for assessing the accuracy of using the C version of the Function Mask (CFMask) in the Landsat Climate Data Records (CDRs) for marsh area estimation. The assessment is performed using the Landsat CDRs and the United States Geological Survey (USGS) Digital Orthophoto Quadrangle (DOQ) collected in Barataria Basin at similar times (within two days) in 1998, 1999, and 2005. For each data set, 100 points are generated randomly within the DOQ food print and stratified by land cover classes.

*Data sets: Landsat CDR 24* ^*th*^ *Feb 1998 (Path 22 Row 40) and DOQ 23* ^*rd*^ *Feb 1998 (DI00000001009761)*			
Class	Predicted Land	Predicted water	User
Actual land	57		1
Actual water	12	31	0.72
Producer	0.83	1	Overall = 0.86
*Data sets: Landsat CDR 10* ^*th*^ *Jan 1999 (Path 22 Row 40) and DOQ 10* ^*th*^ *Jan 1999 (DI00000001063570)*			
Class	Predicted Land	Predicted water	User
Actual land	48	0	1
Actual water	16	36	0.69
Producer	0.75	1	Overall = 0.84
*Data sets: Landsat CDR 25th Oct 2005 (Path 22 Row 40) and DOQ 27* ^*th*^ *Oct 2005 (DI00000100235117)*			
Class	Predicted Land	Predicted water	User
Actual land	42	1	0.97
Actual water	11	46	0.81
Producer	0.79	0.98	Overall = 0.89
*Data sets: Landsat CDR 25th Oct 2005 (Path 22 Row 40) and DOQ 27* ^*th*^ *Oct 2005 (DI00000100235120)*			
Class	Predicted Land	Predicted water	User
Actual land	22	2	0.92
Actual water	6	70	0.92
Producer	0.79	0.97	Overall = 0.92
*Data sets: Landsat CDR 25th Oct 2005 (Path 22 Row 40) and DOQ 27* ^*th*^ *Oct 2005 (DI00000100235121)*			
Class	Predicted Land	Predicted water	User
Actual land	35	2	0.95
Actual water	10	53	0.84
Producer	0.78	0.96	Overall = 0.88
*Data sets: Landsat CDR 25th Oct 2005 (Path 22 Row 40) and DOQ 27* ^*th*^ *Oct 2005 (DI00000100235125)*			
Class	Predicted Land	Predicted water	User
Actual land	32		1
Actual water	5	63	0.93
Producer	0.86	1	Overall = 0.95
*Data sets: Landsat CDR 25th Oct 2005 (Path 22 Row 40) and DOQ 27* ^*th*^ *Oct 2005 (DI00000100235524)*			
Class	Predicted Land	Predicted water	User
Actual land	20	1	0.95
Actual water	5	74	0.94
Producer	0.8	0.99	Overall = 0.94
*Data sets: Landsat CDR 25* ^*th*^ *Oct 2005 (Path 22 Row 40) and DOQ 27* ^*th*^ *Oct 2005 (DI00000100235525)*			
Class	Predicted Land	Predicted water	User
Actual land	30	2	0.94
Actual water	11	57	0.84
Producer	0.73	0.97	Overall = 0.87
Summary	Overall accuracy		0.89 ± 0.04

**Table A4:** Significant correlations (r) between marsh area and phenological changes, and the environmental variables, that is, year, sea‐level (SL), atmospheric CO_2_, temperature (T), precipitation (P), salinity (S), discharge (D), Oceanic Niño Index (ONI) from 1984 to 2014. All the correlations between the variables are tested, but only significant correlations are shown in the table (P < 0.05)

	Year	SL	CO_2_	T	P	S	ONI
Marsh area							
Freshwater							
Intermediate					‐0.5		0.6
Brackish	‐0.8	‐0.8	‐0.7	‐0.9	0.7		
Saline	‐0.6	‐0.5	‐0.6				
Marsh phenology, peak NDVI							
Freshwater							
Marsh phenology, peak NDVI day							
Saline				0.5			
Marsh phenology, growing season length							
Intermediate	0.4		0.4				
Brackish	0.4		0.4				
Saline							

## References

[ece35215-bib-0001] Arp, W. J. , Drake, B. G. , Pockman, W. T. , Curtis, P. S. , & Whigham, D. F. (1993). Interactions between C_3_ and C_4_ salt marsh plant species during 4 years of exposure to elevated atmospheric CO_2_ . Vegetatio, 104, 133–143. 10.1007/bf00048149

[ece35215-bib-0002] Bianchi, T. S. , & Allison, M. A. (2009). Large‐river delta‐front estuaries as natural “recorders'' of global environmental change. Proceedings of the National Academy of Sciences of the United States of America, 106, 8085–8092. 10.1073/pnas.0812878106 19435849 PMC2688901

[ece35215-bib-0003] Boyce, M. S. , Vernier, P. R. , Nielsen, S. E. , & Schmiegelow, F. K. A. (2002). Evaluating resource selection functions. Ecological Modelling, 157, 281–300. 10.1016/s0304-3800(02)00200-4

[ece35215-bib-0004] Chabreck, R. H. , & Linscombe, G. (1988). Vegetative type map of the Louisiana coastal marshes. Baton Rouge, LA: Louisiana Department of Wildlife and Fisheries.

[ece35215-bib-0005] Chabreck, R. H. , & Linscombe, G. (1997). Vegetative type map of the Louisiana coastal marshes. Baton Rouge, LA: Louisiana Department of Wildlife and Fisheries.

[ece35215-bib-0006] Charles, H. , & Dukes, J. S. (2009). Effects of warming and altered precipitation on plant and nutrient dynamics of a New England salt marsh. Ecological Applications, 19, 1758–1773. 10.1890/08-0172.1 19831068

[ece35215-bib-0007] Cherry, J. A. , McKee, K. L. , & Grace, J. B. (2009). Elevated CO_2_ enhances biological contributions to elevation change in coastal wetlands by offsetting stressors associated with sea‐level rise. Journal of Ecology, 97, 67–77. 10.1111/j.1365-2745.2008.01449.x

[ece35215-bib-0008] Climatological Data Publications . National Centers for Environmental Information, National Oceanic and Atmospheric Administration (NOAA). Retrieved from https://www.ncdc.noaa.gov/IPS/cd/cd.html (last access 2019-05-03)

[ece35215-bib-0040] Cold & Warm Episodes by Season . Climate Prediction Center, National Weather Service (NWS). Retrieved from https://origin.cpc.ncep.noaa.gov/products/analysis_monitoring/ensostuff/ONI_v5.php (last access 2019-05-03)

[ece35215-bib-0010] Couvillion, B. R. , Barras, J. A. , Steyer, G. D. , Sleavin, W. , Fischer, M. , Beck, H. , … Heckman, D. (2011). Land area change in coastal Louisiana from 1932 to 2010: U.S. Geological Survey Scientific Investigations Map 3164, scale 1:265,000, 12 p. pamphlet.

[ece35215-bib-0011] Craft, C. (2007). Freshwater input structures soil properties, vertical accretion, and nutrient accumulation of Georgia and U.S. tidal marshes. Limnology and Oceanography, 52, 1220–1230. 10.4319/lo.2007.52.3.1220

[ece35215-bib-0021] Data Distribution Centre . Carbon Dioxide: Projected emissions and concentrations. The Intergovernmental Panel on Climate Change, IPCC. Retrieved from http://www.ipcc-data.org/observ/ddc_co2.html (last access 2019-05-03)

[ece35215-bib-0012] Drake, B. G. (2014). Rising sea level, temperature, and precipitation impact plant and ecosystem responses to elevated CO2 on a Chesapeake Bay wetland: Review of a 28‐year study. Global Change Biology, 20, 3329–3343. 10.1111/gcb.12631 24820033

[ece35215-bib-0013] Duarte, C. M. , & Cebrian, J. (1996). The fate of marine autotrophic production. Limnology and Oceanography, 41, 1758–1766. 10.4319/lo.1996.41.8.1758

[ece35215-bib-0014] Duarte, C. M. , Losada, I. J. , Hendriks, I. E. , Mazarrasa, I. , & Marbà, N. (2013). The role of coastal plant communities for climate change mitigation and adaptation. Adaptation and Mitigation Strategies for Climate Change, 3, 961.

[ece35215-bib-0038] Earth System Research Laboratory , Global Monitoring Division, National Oceanic and Atmospheric Administration (NOAA). Retrieved from https://www.esrl.noaa.gov/gmd/dv/data/ (last access 2019-05-03)

[ece35215-bib-0015] Erickson, J. E. , Megonigal, J. P. , Peresta, G. , & Drake, B. G. (2007). Salinity and sea level mediate elevated CO_2_ effects on C_3_‐C_4_ plant interactions and tissue nitrogen in a Chesapeake Bay tidal wetland. Global Change Biology, 13, 202–215. 10.1111/j.1365-2486.2006.01285.x

[ece35215-bib-0016] Ghannoum, O. (2009). C_4_ photosynthesis and water stress. Annals of Botany, 103, 635–644. 10.1093/aob/mcn093 18552367 PMC2707343

[ece35215-bib-0017] González, J. L. , & Törnqvist, T. E. (2011). Coastal Louisiana in crisis: Subsidence or sea level rise? Eos, Transactions American Geophysical Union, 87, 493–498. 10.1029/2006eo450001

[ece35215-bib-0018] Gosselink, J. G. (1984). The ecology of delta marshes of coastal Louisiana: A community profile. Washington, DC: U. S. Fish and Wildlife Service.

[ece35215-bib-0019] Hardin, J. W. , Hilbe, J. M. , & Hilbe, J. (2007). Generalized linear models and extensions, second edition. College Station, TX: Stata Press.

[ece35215-bib-0020] Hinson, A. L. , Feagin, R. A. , Eriksson, M. , Najjar, R. G. , Herrmann, M. , Bianchi, T. S. , … Boutton, T. (2017). The spatial distribution of soil organic carbon in tidal wetland soils of the continental United States. Global Change Biology, 23, 5468–5480. 10.1111/gcb.13811 28815992

[ece35215-bib-0009] IPCC AR4 (2007). Climate Change 2007: The Physical Science Basis. Contribution of Working Group I to the Fourth Assessment Report of the Intergovernmental Panel on Climate Change Solomon S., Qin D., Manning M., Chen Z., Marquis M., Averyt K. B., Tignor M. and Miller H. L. (Eds.) Cambridge, UK: Cambridge University Press, 996 pp.

[ece35215-bib-0022] Jankowski, K. L. , Törnqvist, T. E. , & Fernandes, A. M. (2017). Vulnerability of Louisiana's coastal wetlands to present‐day rates of relative sea‐level rise. Nature Communications, 8, 14792 10.1038/ncomms14792 PMC5355890 28290444

[ece35215-bib-0023] Kearney, M. S. , & Turner, R. E. (2015). Microtidal marshes: Can these widespread and fragile marshes survive increasing climate–sea level variability and human action? Journal of Coastal Research, 32, 686–699. 10.2112/jcoastres-d-15-00069.1

[ece35215-bib-0024] Kirwan, M. L. , & Blum, L. K. (2011). Enhanced decomposition offsets enhanced productivity and soil carbon accumulation in coastal wetlands responding to climate change. Biogeosciences, 8, 987–993. 10.5194/bg-8-987-2011

[ece35215-bib-0025] Langley, J. A. , McKee, K. L. , Cahoon, D. R. , Cherry, J. A. , & Megonigal, J. P. (2009). Elevated CO_2_ stimulates marsh elevation gain, counterbalancing sea‐level rise. Proceedings of the National Academy of Sciences of the United States of America, 106, 6182–6186. 10.1073/pnas.0807695106 19325121 PMC2661312

[ece35215-bib-0026] Langley, J. A. , & Megonigal, J. P. (2010). Ecosystem response to elevated CO_2_ levels limited by nitrogen‐induced plant species shift. Nature, 466, 96–99. 10.1038/nature09176 20596018

[ece35215-bib-0027] Langley, J. A. , Mozdzer, T. J. , Shepard, K. A. , Hagerty, S. B. , & Megonigal, J. P. (2013). Tidal marsh plant responses to elevated CO_2_, nitrogen fertilization, and sea level rise. Global Change Biology, 19, 1495–1503. 10.1111/gcb.12147 23504873

[ece35215-bib-0028] Linscombe, G. , & Chabreck, R. (2001). Task III.8—Coastwide aerial survey, brown marsh 2001 assessment: Salt marsh dieback in Louisiana. Retrieved from https://lacoast.gov/crms_viewer2/html/ref_vegetation.htm (last access 2019-05-03)

[ece35215-bib-0029] Mariotti, G. , & Fagherazzi, S. (2010). A numerical model for the coupled long‐term evolution of salt marshes and tidal flats. Journal of Geophysical Research‐Earth Surface, 115, F01004 10.1029/2009JF001326

[ece35215-bib-0030] McLeod, E. , Chmura, G. L. , Bouillon, S. , Salm, R. , Björk, M. , Duarte, C. M. , … Silliman, B. R. (2011). A blueprint for blue carbon: Toward an improved understanding of the role of vegetated coastal habitats in sequestering CO_2_ . Frontiers in Ecology and the Environment, 9, 552–560. 10.1890/110004

[ece35215-bib-0031] Mo, Y. , Kearney, M. , Riter, A. , Zhao, F. , & Tilley, D. (2018). Assessing biomass of diverse coastal marsh ecosystems using statistical and machine learning models. International Journal of Applied Earth Observation and Geoinformation, 68, 189–201. 10.1016/j.jag.2017.12.003

[ece35215-bib-0032] Mo, Y. , Momen, B. , & Kearney, M. S. (2015). Quantifying moderate resolution remote sensing phenology of Louisiana coastal marshes. Ecological Modelling, 312, 191–199. 10.1016/j.ecolmodel.2015.05.022

[ece35215-bib-0033] Morris, J. T. , Sundareshwar, P. V. , Nietch, C. T. , Kjerfve, B. , & Cahoon, D. R. (2002). Responses of coastal wetlands to rising sea level. Ecology, 83, 2869–2877. 10.2307/3072022

[ece35215-bib-0034] Mudd, S. M. , D'Alpaos, A. , & Morris, J. T. (2010). How does vegetation affect sedimentation on tidal marshes? Investigating particle capture and hydrodynamic controls on biologically mediated sedimentation. Journal of Geophysical Research‐Earth Surface, 115, F03029 10.1029/2009jf001566

[ece35215-bib-0035] Myneni, R. B. , Keeling, C. D. , Tucker, C. J. , Asrar, G. , & Nemani, R. R. (1997). Increased plant growth in the northern high latitudes from 1981 to 1991. Nature, 386, 698–702. 10.1038/386698a0

[ece35215-bib-0036] Nahlik, A. M. , & Fennessy, M. S. (2016). Carbon storage in US wetlands. Nature Communications, 7, 13835 10.1038/ncomms13835 PMC5159918 27958272

[ece35215-bib-0039] National Water Information System . United States Geological Survey (USGS). Retrieved from https://waterdata.usgs.gov/la/nwis/current/?type=flow (last access 2018-07-24)

[ece35215-bib-0041] Nellemann, C. , & Corcoran, E. Duarte, C. M. , Valdés, L. , De Young, C. , Fonseca, L. , Grimsditch, G. (Eds). (2009). Blue Carbon. A Rapid Response Assessment. United Nations Environment Programme, GRID-Arendal. Retrieved from www.grida.no. (last access 2019-05-03)

[ece35215-bib-0042] Nyman, J. A. , Delaune, R. D. , Roberts, H. H. , & Patrick, W. H. (1993). Relationship between vegetation and soil formation in a rapidly submerging coastal marsh. Marine Ecology Progress Series, 96, 269–279. 10.3354/meps096269

[ece35215-bib-0043] Rasse, D. P. , Peresta, G. , & Drake, B. G. (2005). Seventeen years of elevated CO_2_ exposure in a Chesapeake Bay Wetland: Sustained but contrasting responses of plant growth and CO_2_ uptake. Global Change Biology, 11, 369–377. 10.1111/j.1365-2486.2005.00913.x

[ece35215-bib-0044] Ratliff, K. M. , Braswell, A. E. , & Marani, M. (2015). Spatial response of coastal marshes to increased atmospheric CO2. Proceedings of the National Academy of Sciences of the United States of America, 112, 15580–15584. 10.1073/pnas.1516286112 26644577 PMC4697407

[ece35215-bib-0045] Richards, S. A. (2005). Testing ecological theory using the information‐theoretic approach: Examples and cautionary results. Ecology, 86, 2805–2814. 10.1890/05-0074

[ece35215-bib-0046] Rozema, J. , Dorel, F. , Janissen, R. , Lenssen, G. , Broekman, R. , Arp, W. , & Drake, B. G. (1991). Effect of elevated atmospheric CO_2_ on growth, photosynthesis and water relations of salt marsh grass species. Aquatic Botany, 39, 45–55. 10.1016/0304-3770(91)90021-v

[ece35215-bib-0047] Sasser, C. E. , Visser, J. M. , Mouton, E. , Linscombe, J. , & Hartley, S. B. (2008). Vegetation types in coastal Louisiana in 2007: U.S. Geological Survey Open‐File Report 2008–1224, 1 sheet, scale 1:550,000.

[ece35215-bib-0048] Sasser, C. E. , Visser, J. M. , Mouton, E. , Linscombe, J. , & Hartley, S. B. (2014). Vegetation types in coastal Louisiana in 2013: U.S. Geological Survey Scientific Investigations Map 3290, 1 sheet, scale 1:550,000. 10.3133/sim3290

[ece35215-bib-0049] Schuerch, M. , Spencer, T. , Temmerman, S. , Kirwan, M. L. , Wolff, C. , Lincke, D. , … Brown, S. (2018). Future response of global coastal wetlands to sea‐level rise. Nature, 561, 231–234. 10.1038/s41586-018-0476-5 30209368

[ece35215-bib-0050] Smith, J. M. , Cialone, M. A. , Wamsley, T. V. , & McAlpin, T. O. (2010). Potential impact of sea level rise on coastal surges in southeast Louisiana. Ocean Engineering, 37, 37–47. 10.1016/j.oceaneng.2009.07.008

[ece35215-bib-0051] Stagg, C. L. , Schoolmaster, D. R. , Krauss, K. W. , Cormier, N. , & Conner, W. H. (2017). Causal mechanisms of soil organic matter decomposition: Deconstructing salinity and flooding impacts in coastal wetlands. Ecology, 98, 2003–2018. 10.1002/ecy.1890 28489250

[ece35215-bib-0052] Temmerman, S. , Moonen, P. , Schoelynck, J. , Govers, G. , & Bouma, T. J. (2012). Impact of vegetation die‐off on spatial flow patterns over a tidal marsh. Geophysical Research Letters, 39, L03406 10.1029/2011gl050502

[ece35215-bib-0037] Tides and Currents . National Oceanic and Atmospheric Administration (NOAA). Retrieved from https://tidesandcurrents.noaa.gov/sltrends/sltrends_station.shtml?xml:id=8761724 (last access 2019-05-03)

[ece35215-bib-0053] Turner, R. E. , Baustian, J. J. , Swenson, E. M. , & Spicer, J. S. (2006). Wetland sedimentation from Hurricanes Katrina and Rita. Science, 314, 449–452. 10.1126/science.1129116 16990516

[ece35215-bib-0054] Turner, R. E. , Kearney, M. S. , & Parkinson, R. W. (2017). Sea‐level rise tipping point of delta survival. Journal of Coastal Research, 34, 470–474. 10.2112/JCOASTRES-D-17-00068.1

[ece35215-bib-0055] Van der Putten, W. H. , Macel, M. , & Visser, M. E. (2010). Predicting species distribution and abundance responses to climate change: Why it is essential to include biotic interactions across trophic levels. Philosophical Transactions of the Royal Society of London. Series B, Biological Sciences, 365, 2025–2034. 10.1098/rstb.2010.0037 20513711 PMC2880132

[ece35215-bib-0056] Walther, G.‐R. , Post, E. , Convey, P. , Menzel, A. , Parmesan, C. , Beebee, T. J. C. , … Bairlein, F. (2002). Ecological responses to recent climate change. Nature, 416, 389–395. 10.1038/416389a 11919621

[ece35215-bib-0057] Wang, H. , Yang, Z. , Saito, Y. , Liu, J. P. , Sun, X. , & Wang, Y. (2007). Stepwise decreases of the Huanghe (Yellow River) sediment load (1950‐2005): Impacts of climate change and human activities. Global and Planetary Change, 57, 331–354. 10.1016/j.gloplacha.2007.01.003

[ece35215-bib-0058] Weston, N. B. , Vile, M. A. , Neubauer, S. C. , & Velinsky, D. J. (2011). Accelerated microbial organic matter mineralization following salt‐water intrusion into tidal freshwater marsh soils. Biogeochemistry, 102, 135–151. 10.1007/s10533-010-9427-4

[ece35215-bib-0059] Whiting, G. J. , & Chanton, J. P. (1993). Primary production control of methane emission from wetlands. Nature, 364, 794–795. 10.1038/364794a0

[ece35215-bib-0060] Wu, W. , Huang, H. L. , Biber, P. , & Bethel, M. (2017). Litter decomposition of *Spartina alterniflora* and *Juncus roemerianus*: Implications of climate change in salt marshes. Journal of Coastal Research, 33, 372–384. 10.2112/jcoastres-d-15-00199.1

[ece35215-bib-0061] Zhou, L. , Tucker, C. J. , Kaufmann, R. K. , Slayback, D. , Shabanov, N. V. , & Myneni, R. B. (2001). Variations in northern vegetation activity inferred from satellite data of vegetation index during 1981 to 1999. Journal of Geophysical Research‐Atmospheres, 106, 20069–20083. 10.1029/2000jd000115

[ece35215-bib-0062] Zhu, Z. , & Woodcock, C. E. (2012). Object‐based cloud and cloud shadow detection in Landsat imagery. Remote Sensing of Environment, 118, 83–94. 10.1016/j.rse.2011.10.028

